# Editorial: Clonality in the Anthropocene: adaptation, evolution, and functioning of clonal plants from individuals to ecosystems

**DOI:** 10.3389/fpls.2024.1448891

**Published:** 2024-08-22

**Authors:** Fei-Hai Yu, Jian-Ping Tao, Asad Shabbir

**Affiliations:** ^1^ Institute of Wetland Ecology & Clone Ecology, Zhejiang Provincial Key Laboratory of Evolutionary Ecology and Conservation, Taizhou University, Taizhou, China; ^2^ School of Life Sciences, Southwest University, Chongqing, China; ^3^ Weed Research Unit, Invasive Species Biosecurity, New South Wales Department of Primary Industries, Orange, NSW, Australia

**Keywords:** biological invasions, clonal growth, ecosystem functioning, global change, human activities

In the Anthropocene, plants on Earth face a number of challenges with ongoing global changes in climate, land use, acidity, and toxin concentration. Clonal plants, capable of both asexual (clonal) and sexual reproduction, exhibit a wide distribution and reveal considerable benefits in many habitats, which may be connected with their distinctive characteristics of clonality (Herben and Klimešová, 2020; Roiloa et al., 2023). Clonal traits include clonal regeneration (e.g., production of asexual individuals, called ramets), clonal integration (e.g., resource and signaling sharing between interconnected ramets of the same clone), clonal resource foraging (e.g., selective positioning of ramets), clonal storage and resource reallocation (e.g., reallocation of stored energy/nutrients between interconnected ramets), initiation of meristem bud banks, and trade-offs between clonal and sexual reproduction, which enable clonal plants to quickly adapt to environmental changes (Chen et al., 2019; Klimešová et al., 2021; Shi et al., 2021; Dong et al., 2022). The environmental conditions experienced by a parental ramet of a clonal plant can be transmitted to its offspring via clonal reproduction (i.e., clonal parental effects), thereby influencing the phenotypes of the progeny (Latzel and Klimešová, 2010; Luo et al., 2022; Quan et al., 2022). These adaptations may enhance survival, competitiveness, invasiveness, and the spread of clonal species across ecosystems, in response to global climatic change during the Anthropocene (Wang et al., 2017; 2024; Zhang et al., 2024). Numerous studies have sought to integrate clonality into the research agenda of plant ecology, thereby offering a roadmap to gain valuable insights into the evolutionary dynamics of plants (Cornelissen et al., 2014; Klimešová et al., 2021; Roiloa et al., 2023).

This Research Topic compiles research papers aimed at understanding how clonal plants respond to environmental changes and their contributions to patterns and processes at the population, community, and ecosystem levels. It contributes to enhance our understanding of the roles clonality plays in ecosystem functioning, the influence of clonal growth on the invasiveness of alien plants and the invisibility of native plant communities, as well as the mechanisms, functioning, and adaptive strategies of plant clonality in response to global change at various ecological levels.

Three papers delve into physiological adaptation and effects of exogenous hormone on clonal plants under varying environmental stress conditions. In their study on the stoloniferous herb *Centella asiatica*, Duan et al. discovered that the external application of abscisic acid (ABA) within the clonal network led to a significant increase in biomass accumulation both in the below-ground parts and across the entire clonal fragment of the plant. These findings suggest that the rapid activation and sustained resistance responses induced by local exogenous ABA application within the clonal network may enhance the fitness of clonal plants exposed to abiotic stress. By cultivating a clonal plant *Alternanthera philoxeroides* under different submergence depths, Jing et al. demonstrated that plants achieved greater growth at 2 m and 5 m submergence depths compared to control plants (un-submerged), and gibberellin (GA) induced the differential elongation of the internode as plant submerged at a depth of 2 m had the highest GA accumulation. Therefore, the effects of submergence depth and duration on stem elongation have improved our understanding of the physiological adaptation of clonal plants in deeply flooded environments. In field populations, individual rhizome systems of clonal herb *Podophyllum peltatum* were fed ^14^CO_2_ with different times of demography, Watson and Vuorisalo showed that carbon fixed at various times was utilized differently, with assimilates preferentially moving into old rhizomes rather than supporting the formation of new ramets. These findings suggest that demography influences, integrative physiology, and physiological restrictions on within-season redistribution of assimilates limit the capacities of clonal plants to adapt to rapid environmental changes in the Anthropocene.

Four papers examine physiological integration, resource sharing, and resource utilization of clonal plants across various vegetation environments. In a pot experiment tracing the movement of ^15^N, Zhao et al. found that ^15^N translocation between connected ramets of moso bamboo (*Phyllostachys pubescens*) was determined by the source-sink relationship in heterogeneous environments. The allocation of ^15^N in the fertilized ramet was higher compared to the connected unfertilized ramet. The findings suggest that physiological integration significantly improved the nitrogen use efficiency of moso bamboo, which has implications for fertilization management in moso bamboo forests. Guo et al. demonstrated that the dominant clonal grass *Leymus chinensis* benefited more from physiological integration in sexual reproduction compared to companion clonal species in an *in situ* ^15^N labeling experiment in a grassland. Thus, differences in the ability of physiological integration between the dominant and companion species may explain the dominance of *L. chinensis* in the grassland. Yu et al. found that the performance of a clonal fern *Pyrrosia nudaa* increased with the developmental age of the ramets but decreased with an increasing number of ramets in a clonal fragment. These age-dependent impacts of clonal fragmentation provide insights into the biodiversity conservation of epiphytes and forest management in man-made plantations. Xing et al. showed that neighboring touches on parental ramets of *Glechoma longituba* influenced the performance of offspring ramets, and this effect was depended on the light environment. These results suggest that the interaction between neighboring touch and shade environment influences the growth of understory plants.

One paper investigates the successful invasions of alien clonal plants. Zhang et al. found that connection between ramets had a more pronounced effect on the biomass allocation pattern of alien invasive plants than disconnection, resulting in a greater increase in biomass for invasive plants compared to native plants. These results suggest that invasive clonal plants possess a greater capacity for resource partitioning than native plants, which may confer a competitive advantage and enable them to successfully invade in some heterogeneous habitats, such as forest edges.

Three papers focus on the role of bud banks in clonal plants. Through a chronosequence study of evergreen conifer plantations, Song et al. found that close-to-nature and gap management modes significantly facilitated the diversity of clonal plants, the overall plant diversity of the communities, and various parameters of ecosystem service functions in *Cunninghamia lanceolata* plantations. The diversity of clonal plants was significantly positively correlated with ecosystem service functions following forest management. They suggest that the link between forest management, diversity, and ecosystem functions should be emphasize to elucidate the mechanism of traits-ecosystem functioning relationships and the restoration of degraded plantations. In a bamboo ecosystem, Zou et al. found that the size of clonal fragments of *Phyllostachys bissetii* contributed more to the soil nitrogen turnover process compared to clonal integration, while it had the opposite effect on soil carbon availability. The findings are critical for understanding the nutrient turnover of *P. bissetii* under stressed conditions. Wu et al. discovered that vegetation attributes significantly affected bud banks in wetland ecosystems, while soil properties had a strong influence on bud banks in farmland and alpine meadow ecosystems. These findings suggest that changes in land use can impact the size and composition of bud banks.

Two papers focus on measurement tools, application, and prediction for restoration and conservation of clonal plants. Ohsowski et al. provided a rapid field-based clonal *Typha* identification and biomass assessment tool targeted management for regional conservation of *T. latifolia* and ecological restoration of wetlands impacted by invasive *Typha* taxa. To understand the relationship between plant phenological traits and phylogeny, Shahzad et al. found that phylogeny, growth form, and functional features influenced the diversity of flowering phenology within species in conjunction with local climate circumstances. This understanding aids in comprehending the evolutionary conservation mechanisms of plant phenological traits, including clonal traits, in relation to evolutionary processes across different geographical and climatic zones.

Adaptation, evolution, and functioning of clonal plants have drawn increasing attention in recent years (Klimešová et al., 2021; Roiloa et al., 2023). However, there is still much to explore in this field within the plant ecology agenda. The mechanisms at individual, population, and community scales and their interactions across scales in the Anthropocene, have rarely been discussed so far. We hope to improve and expand our knowledge of this crucial issue in the future.
